# VAP Proteins – From Organelle Tethers to Pathogenic Host Interactors and Their Role in Neuronal Disease

**DOI:** 10.3389/fcell.2022.895856

**Published:** 2022-06-08

**Authors:** Suzan Kors, Joseph L. Costello, Michael Schrader

**Affiliations:** College of Life and Environmental Sciences, Biosciences, University of Exeter, Exeter, United Kingdom

**Keywords:** VAPB, FFAT motif, membrane contact sites, endoplasmic reticulum, pathogen-host interactions, amyotrophic lateral scelerosis, VAPA, peroxisomes

## Abstract

Vesicle-associated membrane protein (VAMP)-associated proteins (VAPs) are ubiquitous ER-resident tail-anchored membrane proteins in eukaryotic cells. Their N-terminal major sperm protein (MSP) domain faces the cytosol and allows them to interact with a wide variety of cellular proteins. Therefore, VAP proteins are vital to many cellular processes, including organelle membrane tethering, lipid transfer, autophagy, ion homeostasis and viral defence. Here, we provide a timely overview of the increasing number of VAPA/B binding partners and discuss the role of VAPA/B in maintaining organelle-ER interactions and cooperation. Furthermore, we address how viruses and intracellular bacteria hijack VAPs and their binding partners to induce interactions between the host ER and pathogen-containing compartments and support pathogen replication. Finally, we focus on the role of VAP in human disease and discuss how mutated VAPB leads to the disruption of cellular homeostasis and causes amyotrophic lateral sclerosis.

## Introduction

VAP was initially cloned from the marine mollusk *Aplysia californica* and named vesicle-associated membrane protein (VAMP)-associated protein of 33 kilodaltons (VAP-33) because of its ability to interact with vesicle fusion protein VAMP (also termed synaptobrevin) (Skehel et al., 1995). Since then, VAP proteins have been identified in all eukaryotic cells and reported to interact with a large number of intracellular proteins (Lev et al., 2008; Kamemura and Chihara, 2019). VAPs are C-tail-anchored (or type II) ER membrane proteins with a central coiled-coil domain and N-terminal major sperm (MSP) domain (∼125 residues), which faces the cytoplasmic side (Nishimura et al., 1999). This N-terminal domain consists of an immunoglobulin-like β-sheet and is named after the *Ascaris suum* protein MSP due to its 22% sequence identity (Bullock et al., 1996; Kaiser et al., 2005). The highly conserved VAP protein family consists in mammals of VAPA and VAPB, including the VAPB splice variant VAPC which lacks both the C-terminal transmembrane domain (TMD) and the coiled-coil domain (Weir et al., 1998; Nishimura et al., 1999). Five additional VAPB splice variants were detected at the mRNA level, although protein levels were undetectable in human tissue lysates by immunoblotting (Nachreiner et al., 2010). VAPA and VAPB share 63% sequence identity, mainly due to similarities in the MSP domain, and a clear functional difference between the paralogues has not been established. Recently, the VAP family was extended with the motile sperm domain-containing proteins 1, 2 and 3 (MOSPD1, MOSPD2 and MOSPD3), which also possess an MSP domain and share binding partners with VAPA/B, though with different affinities (Thaler et al., 2011; Mattia et al., 2018; Cabukusta et al., 2020).

VAP proteins are ubiquitously expressed in mammals (Weir et al., 1998; Nishimura et al., 1999; Skehel et al., 2000), with tissue-specific RNA expression patterns during development (Gabetta et al., 2003). They interact with a wide variety of proteins, imparting them with various functions, including organelle membrane tethering (Costello et al., 2017a), lipid transfer between organelles (Kawano et al., 2006; Ngo and Ridgway, 2009), regulation of calcium homeostasis (De Vos et al., 2012; Lindhout et al., 2019), autophagy (Zhao et al., 2018) and the unfolded protein response (UPR) (Kanekura et al., 2006; Gkogkas et al., 2008). VAP might also have extracellular functions through its cleaved and secreted MSP domain (Deidda et al., 2014), although this has been mainly studied in *C. elegans* and *D. melanogaster* (Tsuda et al., 2008; Han et al., 2012).

In this review, we provide a timely update of VAPA/B binding partners and discuss the role of VAPA/B in maintaining organelle-ER interactions and cooperation. This includes VAP hijacking by viruses and intracellular bacteria to induce interactions between the host ER and pathogen-containing compartments, and the recruitment of host proteins to these sites to support pathogen replication. Finally, we focus on how a mutation in VAPB leads to the disruption of cellular homeostasis, causing amyotrophic lateral sclerosis type 8 (ALS8).

## VAP Binding Partners and the Functions of the Ensuing Complex

### The VAP Interaction

The interaction of VAP with a multitude of diverse proteins means that the VAP proteins are important for many cellular processes, including organelle tethering, lipid transfer, autophagy, ion homeostasis and viral defence. Table 1 provides an overview of the current experimentally confirmed VAP interactors and the proposed functions of the ensuing complex. Many binding partners interact with the MSP domain of VAP via a “two phenylalanines in an acidic tract” (FFAT) motif, which consists of the core consensus sequence ^1^EFFDA-E^7^ flanked by acidic residues (Loewen et al., 2003). In the proposed interaction model, the acidic tract upstream of the core initially binds in a non-specific manner to the basic electropositive surface of the MSP domain followed by a second step, in which the core residues bind to the FFAT-binding site to form a stable complex (Kaiser et al., 2005; Furuita et al., 2010). The FFAT motif can vary in sequence quite considerably, whilst still allowing binding interaction, potentially giving proteins a different affinity for VAP (Murphy and Levine, 2016). Many known VAP interactors contain only one or no phenylalanine in the FFAT motif, and there is some redundancy in the sequence (*see* Table 1). In addition, the acidic tract can vary in length and the number of acidic residues. Interestingly, the VAP family proteins MOSPD1 and MOSPD3 favour motifs with “two phenylalanines in a neutral tract” (FFNT) (Cabukusta et al., 2020). Furthermore, the interaction with VAP can be modulated on multiple levels; for instance, the FFAT-VAP binding can be strengthened and reduced by (de)phosphorylation of the FFAT motif (Kumagai et al., 2014; Johnson et al., 2018; Kirmiz et al., 2018; Di Mattia et al., 2020; Guillén-Samander et al., 2021; Kors et al., 2022). VAP dimerization via the TMD and coiled-coil domains might enhance the recruitment of pre-existing homodimers of FFAT proteins (e.g. OSBP) or bring together two unrelated FFAT-containing proteins, stabilizing the complex (Kaiser et al., 2005; Kim et al., 2010). Additionally, some proteins contain two FFAT motifs, suggesting that the interactor could bind to two VAPs at the same time (e.g. PTPIP51 and ORP3, *see* Table 1). However, not all known VAP binding partners possess a FFAT motif–some proteins bind to the MSP in a different, FFAT-independent way, while other proteins mediate the interaction via their and VAP’s TMD (Table 1).

**TABLE 1:** An overview of experimentally confirmed VAP binding partners in mammalian cells.

Complex	Interaction domain		Localisation^a^	MCS	Physiological role of the VAP complex	Reference
	Binding partner	VAP				
**ACBD4**-VAPB	FFAT motif (score 3.5)	MSP	Peroxisomes (TMD)	Peroxisome-ER	Organelle tethering function	Costello et al. (2017b),
	_173_RDLDSE **VFCDS—LE** QL					Kors et al. (2022)
**ACBD5**-VAPA/B	FFAT motif (score 2.5)	MSP	Peroxisomes (TMD)	Peroxisome-ER	Organelle tethering function, implicated in: peroxisome motility; peroxisome membrane expansion; plasmalogen synthesis; maintenance of cholesterol levels	Costello et al. (2017a),
	_259_SDSDSE **VYCDS—ME** QF					Hua et al. (2017)
α**-Synuclein**-VAPB	?	MSP	Cytosol, nucleus, membranes		Disrupts the **PTPIP51**-VAPB interaction, hence mitochondria-ER MCS (affecting Ca^2+^ exchange)	Paillusson et al. (2017)
**ASNA1**-VAPA/B	FFAT motif (score 2.0)	MSP	Cytosol, ER, nucleus		Mediating interaction with the transmembrane-domain recognition complex (TRC; insertion of tail anchored ER proteins)	Baron et al. (2014)
	_8_WGVEAE **EFEDAPD** VE					
**ATF6**-VAPA/B	?	MSP	ER (TMD), nucleus (cleaved)		Modulates the activity of ATF6-regulated transcription of genes involved in the unfolded protein response (UPR)	Gkogkas et al. (2008)
**CALCOCO1**-VAPA/B	FFAT-motif (score 3.0)	MSP	Nucleus, cytosol	Autophagosome-ER	Acts as ER-phagy receptor for degradation of the tubular ER, via ATG8 interaction	Nthiga et al. (2020)
	_674_DHMDGH **FFFS——T—QD** PF					
**CaSR**-VAPA	FFAT motif (score 2.5)^b^	MSP	Plasma membrane (TMD)	Plasma membrane-ER	Ca^2+^ sensing; near surface CaSR expression	Gorkhali et al. (2021)
	_755_ELEDEI **IFIT——CHE** GS					
**CERT**-VAPA/B	FFAT motif (score 1.0)	MSP	Cytosol, Golgi (PH domain)	Golgi-ER	Ceramide transfer from the ER to the Golgi apparatus, for sphingomyelin synthesis	Kawano et al. (2006),
	_315_SLINEE **EFFDAVE** AA					Saito et al. (2008),
						Kumagai et al. (2014)
**CLN8**-VAPA	?	?	ER (TMD), ER-Golgi intermediate compartment (ERGIC; TMD)		*Possibly*: Ceramide metabolism; endo-lysosomal dynamics	Passantino et al. (2013),
						Adhikari et al. (2019),
						Pesaola et al. (2021)
**CDIP1**-VAPA/B	FFAT motif (score 6.0)	MSP	Endocytic compartments (MMD)		CDIP1-induced cell death	Inukai et al. (2021)
	_180_IPCLIN **DFKDVTH** TC					
**FAF1**-VAPA/B	FFAT motif (score 1.0)	MSP	Nucleus, cytosol		Binding of ubiquitinated proteins; recruiting p97 to the ER membrane (involved in ER-associated protein degradation (ERAD))	Baron et al. (2014)
	_289_SDSDGD **DFEDATE** FG					
**FAPP1**-VAPA/B	C-terminus	?	Golgi (PH domain)	Golgi-ER	Formation of the SAC1-FAPP1-VAP complex – binding of FAPP1 to the PI4P-phosphatase SAC1 promotes the phosphatase activity	Venditti et al. (2019)
**FIP200**-VAPA/B	FFAT motif 1 (score 3.0)	MSP	Cytosol, (pre-) autophagosomal structures, lysosomes, nucleus	Isolation membrane-ER	Formation/stabilization of the **ULK1**/FIP200-WIPI2 complex during isolation membrane expansion for autophagosome formation	Zhao et al. (2018)
	_725_AESPES **DFMS——AVN** EF					
	FFAT motif 2 (score 4.0)					
	_206_ECLTRH **SYRECLG** RL					
**GLTP**-VAPA	FFAT motif (score 3.5)	MSP	Cytosol		*Possibly*: Glycolipid transfer, glucosylceramide sensor	Tuuf et al. (2009),
	_26_AVSHLP **PFFDCLG** SP					Backman et al. (2018)
**HCN2**-VAPA/B	TMD	TMD	ER		Regulation of HCN channel Na^+^/K^+^ pacemaker currents; dendritic localization of HCN2	Silbernagel et al. (2018)
**IFITM3**-VAPA	TMD	TMD and CC	Endosomes, lysosomes, plasma membrane		Preventing the VAPA-**OSBP** association, which induces cholesterol accumulation, inhibiting viral entry	Amini-Bavil-Olyaee et al. (2013)
**JMY**-VAPA	FFAT motif (score 1.5)^b^	MSP	Nucleus, cytoskeleton		*Possibly*: Vesicle based transport	Schlüter et al. (2014)
	_312_ETDDPE **EYYES—LS** EL					
**Kv2.1**-VAPA/B	FFAT motif (score 3.5)	MSP	Plasma membrane (TMD)	Plasma membrane-ER	Kv2 channel clustering; regulating proapoptotic K^+^ currents; phosphatidylinositol homeostasis (via **NIR2** recruitment)	Johnson et al. (2018),
	_584_SMSSID **SFIS——CAT** DF					Kirmiz et al. (2018, 2019),
						Schulien et al. (2020)
**Kv2.2**-VAPA/B	FFAT motif (score 3.0)	MSP	Plasma membrane (TMD)	Plasma membrane-ER	Kv2 channel clustering	Johnson et al. (2018),
	_599_STSSID **SFTS——CAT** DF					Kirmiz et al. (2018)
**MIGA2**-VAPA/B	FFAT motif (score 1.5)	MSP	Mitochondria (TMD), lipid droplets	Mitochondria-ER	Linking reactions of *de novo* lipogenesis in mitochondria to triglyceride production in the ER	Freyre et al. (2019)
	_286_SLTSED **SFFS——ATE** LF					
**NIR1**-VAPB	FFAT motif (score 0.0)	MSP	Plasma membrane (LNS2 domain), cytosol	Plasma membrane-ER	Promoting **NIR2** recruitment for phosphatidylinositol homeostasis	Amarilio et al. (2005),
	_28_VESSDD **EFFDARE** EM					Quintanilla et al. (2022)
**NIR2**-VAPB	FFAT motif (score 0.0)	MSP	Golgi (LNS2 domain), plasma membrane (LNS2 domain), cytosol	Golgi-ER	Phosphatidylinositol transfer to the Golgi apparatus and phosphatidylcholine transfer to the ER (important for **CERT** and **OSBP** Golgi targeting/function)	Amarilio et al. (2005),
	_343_ENSSEE **EFFDAHE** GF			Plasma membrane-ER	Phosphatidylinositol transfer from the ER to the plasma membrane	Peretti et al. (2008),Chang et al. (2013),Chang and Liou (2015),Kirmiz et al. (2019)
**NIR3**-VAPB	FFAT motif (score 0.0)	MSP	Plasma membrane (LNS2 domain), cytosol	Plasma membrane-ER	Phosphatidylinositol transfer from the ER to the plasma membrane	Amarilio et al. (2005),
	_338_DESSDD **EFFDAHE** DL				Microtubule interaction	Chang and Liou, (2015)
**OSBP**-VAPA	FFAT motif (score 0.0)_352_DEDDEN **EFFDAPE** II	MSP	Golgi (PH domain), endosomes (PH domain), lysosomes (PH domain), cytosol	Golgi-EREndosome-ERLysosome-ER	Cholesterol transfer from the ER to the Golgi apparatus in exchange for PI4PRegulation of PI4P levels on endosomesCholesterol transfer from the ER to lysosomes, regulating mTORC1 activation	Wyles et al. (2002),Loewen et al. (2003),Mesmin et al. (2013),Dong et al. (2016),Lim et al. (2019)
**ORP1L**-VAPA/B	FFAT motif (score 1.5)	MSP	Late endosomes/lysosomes (PH domain and ankyrin motif), autophagosome, phagolysosome	Late endosome/lysosome (LEL)-ER	Cholesterol transport from the LEL to the ER (high cholesterol) and vice versa (low cholesterol); endosome positioning	Rocha et al. (2009),
	_469_SILSED **EFYDALS** DS			Autophagosome-ER	Regulating autopagosome transport and maturation	Eden et al. (2016),
				Phagolysosome-ER	PI4P transfer to the ER, for phagolysosome resolution	Wijdeven et al. (2016),
						Zhao and Ridgway (2017),
						Levin-Konigsberg et al. (2019)
**ORP2**-VAPA	FFAT motif (score 1.5)	MSP	Lipid droplets, plasma membrane, cytosol	Lipid droplet-ER	Triglyceride metabolism	Weber-Boyvat et al. (2015b)
	_1_MNGEE **EFFDAVT** GF					
**ORP3**-VAPA	FFAT motif 1 (score 1.0)	MSP	Plasma membrane (PH domain), cytosol	Plasma membrane-ER	Stimulating R-Ras signalling	Lehto et al. (2005),
	_444_ITDSLS **EFFDAQE** VL			Late endosome-nuclear envelope	The nuclear transfer of extracellular vesicle-derived materials	Weber-Boyvat et al. (2015a),
	FFAT motif 2 (score 4.5)					Santos et al. (2018)
	_155_FPHEVN **HFFS——GST** IT					
**ORP4L**-VAPA	FFAT motif (score 0.0)	MSP	Golgi (PH domain), plasma membrane	Plasma membrane-ER^c^	Controlling the localization and activation of the phosphoinositide phospholipases C β3 (PLCβ3) at the plasma membrane	Pan et al. (2018),
	_445_EEDEDT **EYFDAME** DS			Golgi-ER	The maintenance of Golgi structure	Pietrangelo and Ridgway, (2018)
**ORP6**-VAP	FFAT motif (score 1.0)^b^	MSP	Plasma membrane (PH domain), cytosol	Plasma membrane-ER	PI4P turnover	Wyles and Ridgway (2004),
	_488_MSESVS **EFFDAQE** VL					Mochizuki et al. (2018)
**ORP7**-VAP	FFAT motif (score 1.0)^b^	MSP	Plasma membrane (PH domain), cytosol		?	Wyles and Ridgway (2004),
	_396_LADSHT **EFFDACE** VL					Weber-Boyvat et al. (2015b)
**ORP9L**-VAPA	FFAT motif (score 1.0)	MSP	Golgi (PH domain), cytosol	Golgi-ER	Golgi organization and protein transport; cholesterol transfer	Wyles and Ridgway (2004),
	_294_YSSSED **EFYDADE** FH					Ngo and Ridgway, (2009)
**PP2Cϵ**-VAP	TMD	TMD	ER (TMD)		Dephosphorylating **CERT**, which promotes Golgi localisation of CERT and enhances the CERT-VAPA interaction	Saito et al. (2008)
**PRA1**-VAP	FFAT motif (score 5.5)^b^	MSP	Golgi (TMD), ER (TMD)	Mitochondria-ER	ER retention of PRA1	Abu Irqeba and Ogilvie, (2020)
	_66_RLVRNV **EYYQS—NY** VF					
**Prestin**-VAPA	?	?	Plasma membrane (TMD)		Prestin translocation to the plasma membrane	Sengupta et al. (2010)
**Protrudin**-VAPA/B	FFAT motif (score 1.0)	MSP, TMD	ER (TMD), endosomes (FYVE domain), plasma membrane (FYVE domain)	Endosome-ER	Endosome trafficking; stimulating process/neurite formation	Saita et al. (2009),
	_280_EAEPDE **EFKDAIE** ET					Matsuzaki et al. (2011),
						Raiborg et al. (2015),
						Petrova et al. (2020)
**PTPIP51**-VAP	FFAT motif 1 (score 3.0)	MSP	Mitochondria (TMD)	Mitochondria-ER	Ca^2+^ delivery to mitochondria from ER stores, which regulates autophagy and synaptic function; phosphatidic acid transfer, important for mitochondrial cardiolipin synthesis	De Vos et al. (2012),
	_151_STGSSS **VYFT——ASS** GA					Stoica et al. (2014),
	FFAT motif 2 (score 3.5)					Gómez-Suaga et al. (2017),
	_160_TASSGA **TFTDAES** EG					Gómez-Suaga et al. (2019),
						Yeo et al. (2021)
**RAB3GAP1**-VAPA/B	FFAT motif (score 0.5)	MSP	Cytosol		Regulating nuclear envelope formation through ERGIC	Baron et al. (2014),
	_578_WSDSEE **EFFECLS** DT					Hantan et al. (2014)
**SCRN1**-VAPA/B	FFAT motif (score 2.5)	MSP	Cytosol		Modulating Ca^2+^ homeostasis and synaptic vesicle cycling; ER dynamics	Lindhout et al. (2019)
	_394_AEVGDL **FYDCVD** TE					
**SNX2**- VAPB	FFAT motif 1 (score 3.5)	MSP	Endosomes (PX domain)	Endosome-ER	Retromer-/WASH-dependent actin nucleation (vesicle budding) of endosomes, with a role of PI4P (*see* **OSBP**)	Dong et al. (2016)
	_21_LEDGED **LFTS——T—VS** TL					
	FFAT motif 2 (score 2.0)					
	_66_DDDRED **LFAEATE** EV					
**STARD3**-VAPA/B	FFAT motif (score 5.5)	MSP	Late endosomes (TMD)	Late endosome-ER	Cholesterol transport from the ER to endosome	Alpy et al. (2013),
	_200_GALSEG **QFYS——PPE** SF					Wilhelm et al. (2017),
						Di Mattia et al. (2020)
**STARD3NL**-VAPA/B	FFAT motif (score 5.5)	MSP	Late endosomes (TMD)	Late endosome-ER	Formation of endosomal tubules	Alpy et al. (2013)
	_201_GGLSDG **QFYS——PPE** SE					
**TRPC3**-VAPB	FFAT motif (score 5.0)	MSP	Plasma membrane	Plasma membrane-ER	Controls TRPC3’s Ca^2+^ current and its receptor-mediated activation	Liu et al. (2022)
	_140_QELQDD **DFYAYDE** DG					
**TTC39B**-VAPB	FFAT motif (score 2.0)	MSP	Cytosol		Stabilizing ER-membrane protein SCAP, involved in hepatic lipogenic gene expression	Hsieh et al. (2021)
	_76_LEADED **VFEDALE** TI					
**ULK1**-VAPA/B	FFAT motif 1 (score 5.5)	MSP	Cytosol, pre-autophagosomal structures	Isolation membrane-ER	Formation/stabilization of the ULK1/**FIP200**-WIPI2 complex during isolation membrane expansion for autophagy	Zhao et al. (2018)
	_87_SVYLVM **EYCNGGD** LA					
	FFAT motif 2 (score 5.5)					
	_74_NIVALY **DFQEMAN** SV					
**Viperin**-VAPA	C-terminus	C-terminus	ER, lipid droplets		Restricting Hepatitis C virus replication complex formation by promoting degradation of viral NS5A through VAPA	Wang et al. (2012),
						Ghosh et al. (2020)
**VPS13A**-VAPA/B	FFAT motif (score 1.0)	MSP	Mitochondria (ATG homology region, PH domain), lipid droplets (PH domain)	Mitochondria-ER	Mitochondria elongation; glycerolipid transfer between membranes	Kumar et al. (2018),
	_836_EDDSEE **EFFDAPC** SP			Lipid droplet-ER	Lipid droplet size and motility; glycerolipid transfer between membranes	Yeshaw et al. (2019)
**VPS13C**-VAPB	FFAT motif (score 0.0)	MSP	Late endosomes/lysosomes (WD40 module), lipid droplets (PH domain)	Endolysosome-ER	Glycerolipid transfer between membranes	Kumar et al. (2018)
	_871_ESESDD **EYFDAED** GE			Lipid droplet-ER	Glycerolipid transfer between membranes	
**VPS13D**-VAPB	FFAT motif (score 5.0)	MSP	Golgi, mitochondria	Mitochondria-ER	Bridging the organelle membranes via MIRO at the mitochondrial membrane (likely similar with peroxisomes); membrane lipid transfer	Guillén-Samander et al. (2021)
	_761_TQFSDD **EYKT——PLA** TP					
**WDR44**-VAPA/B	FFAT motif (score 0.5)	MSP	Cytosol, endosomes, Golgi		Tubular endosome formation and/or stabilization	Baron et al. (2014),
	_3_SESDTE **EFYDAPE** DV					Häsler et al. (2020)
**YIF1A**-VAPB	TMD	MSP	ER-Golgi intermediate compartment (ERGIC; TMD); ER (TMD), Golgi (TMD)		Controls the shuttling of YIF1A between the ERGIC and the ER; promotes intracellular membrane delivery into dendrites	Kuijpers et al. (2013b)

FFAT motif scores were calculated using the FFAT scoring algorithm (best FFAT motif scores zero)^c^(Murphy and Levine, 2016). Phosphorylation of serine/threonine at position 4 (double underlined) of the core (bold) of Phospho-FFAT motifs is critical for VAP binding (Di Mattia et al., 2020). Phosphorylation of serine/threonine at position 5 (underlined) of FFAT motifs abolishes VAP binding (Kors et al., 2022). The cellular localisations of the binding partners are listed. The physiological role describes the function of the VAP complex (binding partners may also have been implicated in other non-VAP related processes, or functions might not have been directly linked to VAP yet). ACBD4/5, acyl-CoA-binding domain-containing protein 4/5; ASNA1 (TRC40), arsenite-stimulated ATPase; ATF6, activating transcription factor 6; CALCOCO1, calcium-binding and coiled-coil domain-containing protein 1; CaSR, calcium-sensing receptor; CERT, ceramide transfer protein; CLN8, ceroid-lipofuscinosis neuronal protein 8; CDIP1, cell death-inducing p53-target protein 1; FAF1, FAS-associated factor 1 (ubiquitin-binding protein); FAPP1, phosphatidylinositol-four-phosphate adapter protein 1; FIP200, FAK family kinase-interacting protein of 200 kDa; GLTP, glycolipid transfer protein; HCN2, hyperpolarization-activated cyclic nucleotide-gated channel 2; IFITM3, interferon-inducible transmembrane protein 3; JMY, junction-mediating and -regulatory protein; Kv2, potassium voltage-gated channel subfamily B; MCS, membrane contact site; MIGA2, mitoguardin 2; MMD, monotopic integral membrane domain; MSP, major sperm protein; NIR, PYK2 N-terminal domain-interacting receptor; ORP, oxysterol-binding protein-related protein; OSBP, oxysterol-binding protein; PP2Cϵ, protein phosphatase 2Cϵ; PRA1, prenylated Rab acceptor 1; PTPIP51, protein tyrosine phosphatase-interacting protein 51; RAB3GAP1, RAB3 GTPase-activating protein catalytic subunit; SCRN1, secernin-1; SNX2, sorting nexin-2; STARD3, StAR-related lipid transfer protein 3; STARD3NL, STARD3 N-terminal like; TMD, transmembrane domain; TRPC3, transient receptor potential channel 3; TTC39B, tetratricopeptide repeat domain containing protein 39 B; ULK1, UNC-51-like autophagy-activating kinase 1; Viperin, Virus inhibitory protein, endoplasmic reticulum-associated, interferon-inducible; VPS13, Vacuolar protein sorting-associated protein 13; WDR44, WD repeat-containing protein 44; YIF1A, YIP1-interacting factor homologue A.

a Due to their interaction with VAP, the proteins also localise at the ER (ER is only mentioned if the protein contains another ER targeting domain, e.g. TMD).

b Predicted FFAT motif, but not confirmed.

c The FFAT score does not indicate the definite binding strength.

### Organelle Tethering

Organelles form membrane contact sites (MCS) for efficient cooperation (Silva et al., 2020). These MCS are mediated by tethering proteins that cross the two opposing membranes, bringing them in close proximity. Various proteins are attracted to these sites to fulfil and regulate specific functions, e.g. membrane lipid and calcium (Ca^2+^) transfer between the organelles. In this section, we will describe the organelle tethering function of VAP in more detail, focussing on the FFAT motif-containing binding partners PTPIP51 and ACBD5 as examples. However, other VAP interactors and functions–many of which relate to MCS–are known. There is an abundance of processes involving VAP at other organelle-ER contacts (Table 1), e.g. CERT transfers ceramide from the ER to the Golgi apparatus (Kawano et al., 2006); STARD3 transfers cholesterol from the ER to endosomes (Wilhelm et al., 2017; Di Mattia et al., 2020); NIR2 transfers phosphatidylinositol from the ER to both the Golgi and plasma membrane, and phosphatidylcholine from the Golgi to the ER (Peretti et al., 2008; Chang and Liou, 2015); while the interaction of VAP with potassium (K^+^) channel Kv2 at plasma membrane-ER contacts is important for Kv2 channel clustering and regulation of K^+^ currents (Johnson et al., 2018; Kirmiz et al., 2018; Schulien et al., 2020). Furthermore, other examples will be discussed in the sections on pathogens and amyotrophic lateral sclerosis type 8 (ALS8).

**FIGURE 1:**
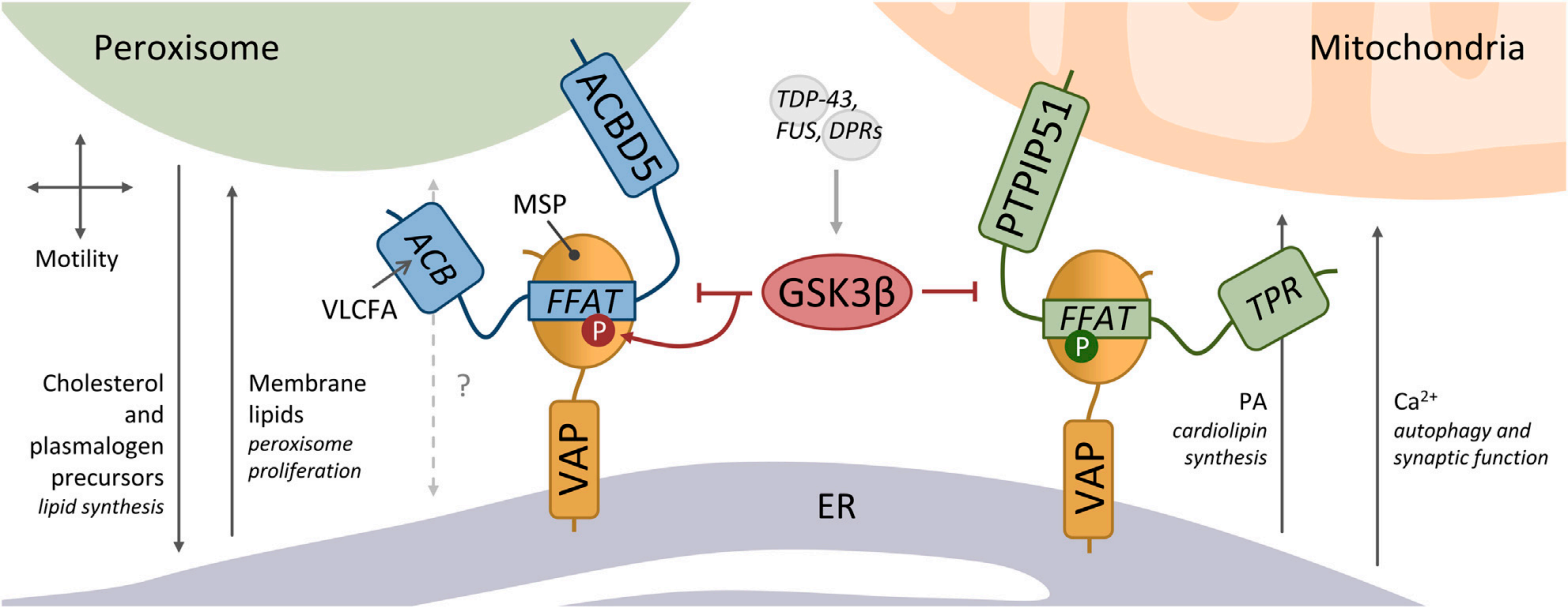
Peroxisome-ER and mitochondria-ER membrane contacts tethered by VAP. ACBD5 interacts via its FFAT motif to the major sperm (MSP domain) of VAP to mediate peroxisome-ER contacts. These peroxisome-ER contacts have been implicated in peroxisome motility, the transfer of cholesterol and plasmalogen precursors for further synthesis in the ER, and the transfer of membrane lipids for peroxisome proliferation. ACBD5 has an acyl-CoA binding (ACB) domain which likely binds very long chain fatty acids (VLCFA). PTPIP51 also binds to the VAP-MSP domain via a FFAT motif, which mediates mitochondria-ER contacts. PTPIP51 has a tetratricopeptide repeat (TPR) domain with which it can bind and transfer phosphatidic acid (PA) to the mitochondria - required for the synthesis of cardiolipin. Ca^2+^ uptake by mitochondria from ER stores at these contacts modulates autophagosome formation and synaptic activity. GSK3β negatively regulates both peroxisome-ER and mitochondria-ER associations. GSK3β acts on the ACBD5-VAP tether by directly phosphorylating the serine residue (S) at position 5 of the ACBD5 FFAT core (^1^VYCDSME^7^). GSK3β can be activated by the ALS-associated proteins TDP-43, FUS and C9orf72-derived dipeptide repeat polypeptides (DPR). Phosphorylation of PTPIP51 at position 4 of the FFAT core (^1^VYFTASS^7^) is critical for binding to VAP.

The membrane proteins PTPIP51 (also named RMDN3) and ACBD5 interact with VAPB, mediating mitochondria-ER and peroxisome-ER associations, respectively (Figure 1) (De Vos et al., 2012; Costello et al., 2017a; Hua et al., 2017). Knockdown of PTPIP51 or ACBD5 reduced the contacts between the respective organelle and the ER, while overexpression increased the associations (Stoica et al., 2014; Costello et al., 2017a). Whilst VAP itself does not appear to possess lipid binding capacity, many of its interacting partners have lipid binding properties, including PTPIP51 and ACBD5. PTPIP51 has a tetratricopeptide repeat (TPR) domain with which it can bind and transfer phosphatidic acid (PA) (Ito et al., 2021; Yeo et al., 2021). PA supply to mitochondria from the ER is required for the synthesis of cardiolipin, an important phospholipid of the inner mitochondrial membrane, which was decreased upon depletion of PTPIP51. This function was independent of the tethering function of PTPIP51 (Yeo et al., 2021). Mitochondria-ER contacts are also important for cellular Ca^2+^ homeostasis, with transport between the organelles mediated by the IP3R-GRP75-VDAC1 complex. Although not directly involved in Ca^2+^ transfer, the PTPIP51-VAPB interaction plays an important tethering role to allow the Ca^2+^ uptake by mitochondria from ER stores (De Vos et al., 2012). This PTPIP51-VAPB-regulated Ca^2+^ delivery modulates autophagosome formation and synaptic activity (Gómez-Suaga et al., 2017, 2019). Additionally, PTPIP51 was shown to be involved in the mitochondrial Ca^2+^ overload during cardiac ischemia/reperfusion, by increasing the mitochondria-sarcoplasmic reticulum contacts (Qiao et al., 2017).

ACBD5 has an acyl-CoA binding (ACB) domain, which has been shown to have lipid/fatty acid binding capacity *in vitro* (Yagita et al., 2017), but it is not yet clear if it directly transfers lipids between peroxisomes and the ER. However, as ACBD5 deficient patients present with accumulation of very long chain fatty acids (VLCFA), it is suggested that ACBD5 facilitates VLCFA transport into peroxisomes for degradation via the peroxisomal ABC transporter for VLCFA (Ferdinandusse et al., 2017; Yagita et al., 2017; Herzog et al., 2018). The ACBD5-VAPB mediated peroxisome-ER contacts have also been implicated in the regulation of peroxisome motility and positioning, and the delivery of lipids for peroxisomal membrane expansion to maintain peroxisome biogenesis (Figure 1) (Costello et al., 2017a; Hua et al., 2017; Darwisch et al., 2020). ACBD5 and VAPB are also required to support the transfer of plasmalogen precursors, of which the synthesis is initiated in peroxisomes and completed in the ER, and for the maintenance of cholesterol levels (Hua et al., 2017; Herzog et al., 2018).

The examples above illustrate some of the various processes that occur at mitochondria-ER and peroxisome-ER contact sites. These processes appear to require contacts to be in a dynamic equilibrium, with reduced contacts reducing the required substrate transfer but increased contacts potentially also resulting in an excess of exchange. For example, whilst loss of PTPIP51-VAPB stimulates autophagy, increased PTPIP51-VAPB inhibits autophagy implying that dynamism in the mitochondria-ER interaction is required for this process (Gómez-Suaga et al., 2017)). In a similar way, whilst loss of ACBD5-VAPB tethering appears to limit peroxisomal membrane expansion, increased ACBD5 levels lead to peroxisomal elongation, potentially implying an excess membrane expansion (Costello et al., 2017a; Kors et al., 2022). Overall, this suggests that these organelle interactions involving VAP protein tethers are highly regulated. One way to regulate tethers would be to modulate the level of interaction between VAP and its interaction partners. In line with this, we revealed that the ACBD5-VAPB tether can be modulated by phosphorylation of serine/threonine residues within the acidic tract of the FFAT motif (Kors et al., 2022), a mechanism initially described for CERT (Kumagai et al., 2014). Phosphorylation of these residues mimics the canonical aspartic and glutamic acid residues, supporting the acidic environment and enhancing binding to VAPB. Notably, the acidic tract of PTPIP51 is also mainly composed of serine/threonine residues, suggesting that phosphorylation of these residues could as well modulate the binding of PTPIP51 to VAPB. Indeed, *in vitro* studies with PTPIP51 FFAT peptide and VAPB protein revealed a low affinity suggesting a minor contribution to mitochondria-ER tethering (Yeo et al., 2021). Although this may be different *in vivo*, phosphorylation of the acidic tract and the FFAT core of PTPIP51 (*see* below) could stengthen the interaction.

In addition to the acidic tract, phosphorylation of the core FFAT motif of both PTPIP51 and ACBD5 also regulates their interaction to VAPB. However, the different positions of the phosphorylated residues have opposing effects on the binding. Phosphorylation of PTPIP51 at position 4 of the FFAT core (^1^VYFTASS^7^) is critical for VAPB binding *in vitro* (Di Mattia et al., 2020), while phosphorylation of ACBD5 at position 5 of the FFAT core (^1^VYCDSME^7^) abolishes the interaction with VAPB (Kors et al., 2022). The canonical FFAT motif possesses aspartic acid (D) at position 4, which could be mimicked by phosphorylated threonine (T) at this position in PTPIP51 to enhance the VAPB binding. The residue at position 5 of the FFAT core–alanine (A) in the canonical motif–binds the VAP MSP domain in a hydrophobic pocket (Kaiser et al., 2005; Furuita et al., 2010). Adding a phosphate group to the serine (S) at this position in ACBD5 likely causes steric hindrance, blocking the interaction. We recently showed that GSK3β can directly phosphorylate this serine residue of the ACBD5 FFAT core (Figure 1). Accordingly, increased GSK3β activity inhibited the ACBD5-VAPB interaction and hence peroxisome-ER contacts, while reduced GSK3β activity increased the organelle associations (Kors et al., 2022). Interestingly, GSK3β also negatively regulates the PTPIP51-VAPB interaction and mitochondria-ER associations, although the precise mechanism is not known (Stoica et al., 2014, 2016; Gómez-Suaga et al., 2022). It was shown that the ALS-associated proteins TDP-43, FUS and C9orf72-derived dipeptide repeat polypeptides (DPR) activate GSK3β, causing disruption of the mitochondria-ER tether and membrane contacts. This suggests altered mitochondria-ER and peroxisome-ER MCS in TDP-43/FUS/C9orf72-induced pathologies.

Overall, there are three regulation mechanisms involving phosphorylation of FFAT motifs: *1*) phosphorylation of residues in the acidic tract enhances the interaction with VAP, acting as a potential fine-tuning mechanism (Kumagai et al., 2014; Di Mattia et al., 2020; Kors et al., 2022); *2*) phosphorylation of S/T in position 4 acts as a switch mechanism (OFF/ON), being critical for VAP binding and defines the so-called “Phospho-FFAT motif” (Kirmiz et al., 2018; Di Mattia et al., 2020; Guillén-Samander et al., 2021); and *3*) phosphorylation of S/T in position 5 also acts as a switch mechanism (ON/OFF), but in this case the phosphorylated FFAT motif is not able to interact with VAP (Mikitova and Levine, 2012; Kors et al., 2022).

## VAP Hijacking by Viruses and Bacteria

The VAP proteins are exploited by various viruses and intracellular bacteria for their replication. Some pathogens hijack VAP via FFAT motif mimicry, while others express pathogenic proteins that interact with VAP or VAP-interactors in other ways. Below we describe how different viruses and bacteria make use of the many functions of VAP for membrane remodelling, the formation of MCS between the host ER and pathogen-containing compartments, and targeting host MCS components to rewire the host lipid metabolism and other processes.

### FFAT Motif-Containing Pathogenic Proteins

#### Chlamydia trachomatis

**FIGURE 2:**
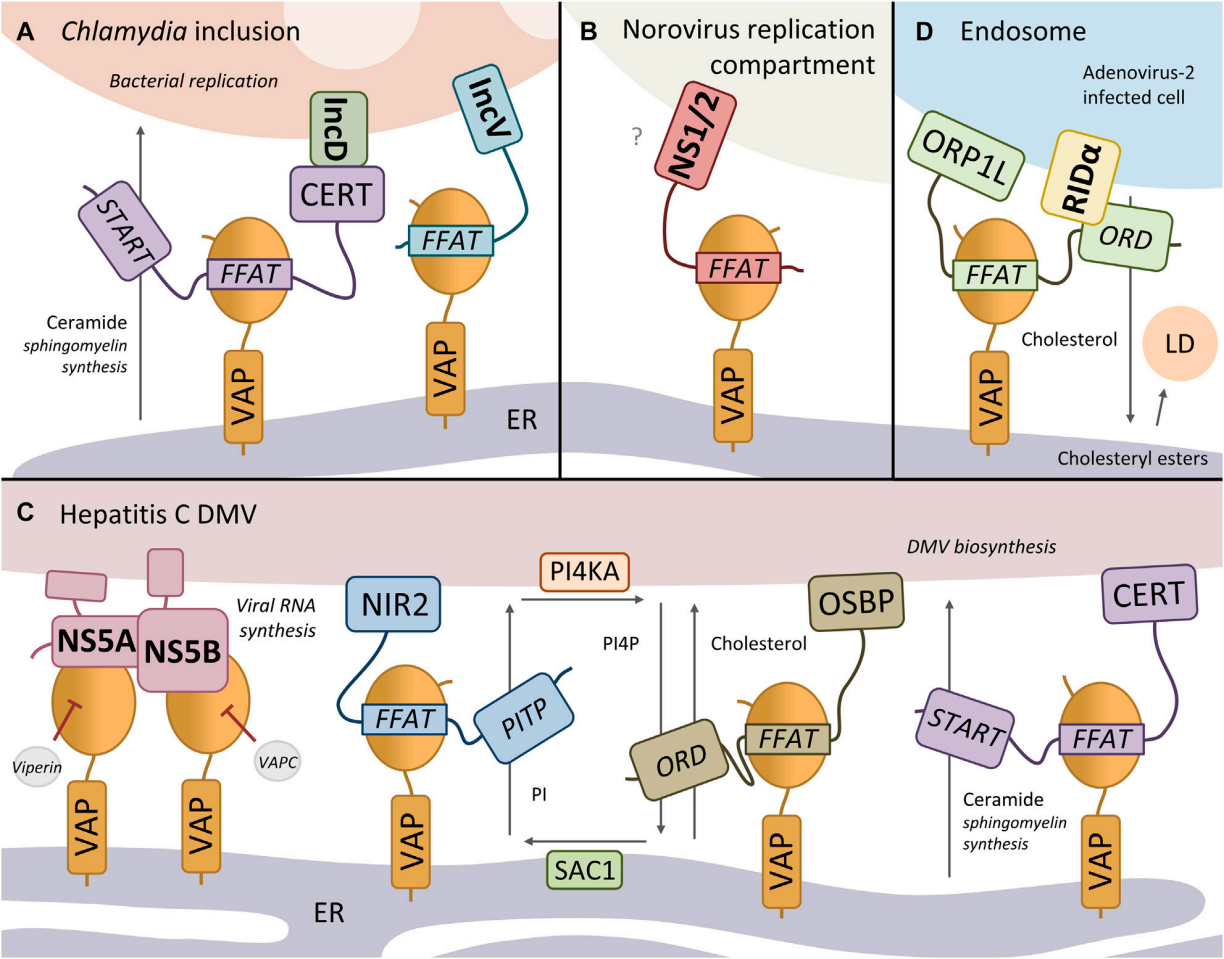
VAP hijacking by bacteria and viruses. **(A)** The Chlamydia integral inclusion membrane proteins IncV and IncD are both found in a complex with VAP. While IncV binds VAP directly via a FFAT motif, IncD interacts with VAP-interactor CERT, forming an IncD-CERT-VAP complex at inclusion-ER contact sites. CERT may facilitate ceramide transfer from the ER to the inclusion membrane for the synthesis of sphingomyelin, important for *C. trachomatis* replication. **(B)** The murine norovirus protein NS1/2 binds VAP via its FFAT motif, critical for viral replication. The NS1/2-VAP may tether the replication membrane to the ER. **(C)** VAP binds to the hepatitis C virus proteins NS5A and NS5B, important for viral RNA replication at double-membrane vesicles (DMVs). VAP supports the viral replication by recruiting host NIR2, OSBP, CERT to the DMV-ER contact site. NIR2 transfers PI from the ER membrane to the DMV membrane, where it is converted to PI4P by the PI4P-kinase PI4KA. The PI4P is then exchanged for cholesterol from the ER by OSBP. The PI4P-phosphatase SAC1 converts PI4P back to PI. CERT transports ceramide from the ER to the DMV, where it is converted to sphingomyelin, important for the biosynthesis of DMVs. **(D)** The Adenovirus-2 protein RIDα directly interacts and recruits ORP1L to maturing early endosomes to form endosome-ER MCS via ORP1L-VAP binding, which facilitates the transport of cholesterol from endosomes to the ER. Here, cholesterol is converted into cholesteryl esters, which are stored in lipid droplets (LD). Bacterial/viral proteins are indicated in bold.

The bacterium *Chlamydia trachomatis* is an intracellular pathogen, causing non-congenital blindness, and is the most common sexually transmitted infection worldwide. The bacterium proliferates inside the cell in a membranous compartment, called an inclusion. The integral inclusion membrane protein IncV has been found to directly interact with VAPA/B via two FFAT motifs upon *C. trachomatis* infection (_280_DSSSSS EYMDALE TV; _256_ESSSSS SFHTPPN SD; Figure 2A) (Stanhope et al., 2017). Overexpression of IncV in *C. trachomatis*-infected cells enhanced the recruitment of VAPA and the ER to the inclusion membrane, suggesting that IncV promotes the formation of inclusion-ER MCS. However, depletion of IncV had only a moderate impact on VAPA enrichment at the inclusion membrane, suggesting that other proteins contribute to the stability of inclusion-ER MCS (e.g. IncD-CERT-VAPA/B complex, *see* below). The two FFAT motifs of IncV both have an acidic tract consisting of multiple serine residues, suggesting that, like ACBD5, its interaction with VAPA/B could be regulated by phosphorylation of these residues, to mimic the negative charge of the conventional acidic residues (Stanhope et al., 2017). The exact contribution of the IncV-VAP interaction in *Chlamydia* pathogenesis remains to be determined.

In mammalian cells, ceramide is transported from the ER to the Golgi complex at MCS by transport protein CERT for the synthesis of the membrane lipid sphingomyelin (Table 1). Another *C. trachomatis* integral inclusion membrane protein, IncD, has been found to interact with host CERT, recruiting CERT and thus its binding partner VAPA/B to the inclusion membrane (Figure 2A) (Derré et al., 2011; Agaisse and Derré, 2014; Kumagai et al., 2018). In this way the IncD-CERT-VAPA/B complex may facilitate ceramide transfer from the ER to the inclusion membrane, where it is converted to sphingomyelin with the use of host and/or bacterial sphingomyelin synthases (Elwell et al., 2011; Tachida et al., 2020). This CERT-dependent sphingomyelin pathway is critical for *C. trachomatis* replication.

Another example of how *C. Trachomatis* hijacks components usually present at host ER-organelle MCS is STIM1. This ER-resident Ca^2+^ sensor protein colocalized with VAPB at inclusion-ER MCS (Agaisse and Derré, 2015). However, the plasma membrane Ca^2+^ channel ORAI1, the interaction partner of STIM1 at PM-ER MCS, did not associate with the inclusion membrane. Instead, STIM1 may work with another Ca^2+^ channel: IP3R, an ER protein present at mitochondria-ER MCS. IP3R has been found to bind both STIM1 (Santoso et al., 2011) and the inclusion membrane protein MrcA, presumably forming a Ca^2+^ signalling complex at the inclusion-ER MCS (Nguyen et al., 2018). Both STIM1 and ITPR3 are required for chlamydial release via extrusion of the inclusion. The regulation of local Ca^2+^ levels may influence the myosin motor complex, which promotes the extrusion.

Overall, the interaction of *Chlamydia* membrane protein IncV with VAPA/B promotes the formation of inclusion-ER MCS within cells. At these sites, *C. trachomatis* redirects several host proteins for sphingomyelin synthesis (e.g. CERT via IncD; important for bacterial replication) and Ca^2+^ signalling (e.g. STIM1 via MrcA; bacterial extrusion) to assist its pathogenicity.

#### Norovirus

Noroviruses are non-enveloped RNA viruses and the primary cause of gastroenteritis. The murine and human (GI) norovirus protein NS1/2 has been reported to interact with VAPA/B (Figure 2B) (Ettayebi and Hardy, 2003; McCune et al., 2017). Structural analysis revealed that the murine NS1/2-VAP interaction is mediated by a FFAT-motif mimic located in the N-terminal NS1 domain of NS1/2 (_40_ESEDEV NYMTPPE QE) (McCune et al., 2017). The FFAT-motif is conserved across murine norovirus strains, although the inherently disordered NS1 domain itself is not well conserved in contrast to the NS2 domain (Baker et al., 2012). Interestingly, NS1/2 has been found to form dimers, a property of many FFAT motif-containing proteins, which could stabilize the interaction with VAP-dimers. It would be interesting to determine whether the human NS1/2^GI^-VAP interaction is also mediated via a FFAT motif.

Strikingly, mutagenesis of the NS1/2 FFAT residues critical for VAP binding eliminated virus replication (McCune et al., 2017). Additionally, VAPA depletion in cells showed that VAPA was important in the early stage of norovirus replication. However, it is not clear how the NS1/2-VAP interaction contributes to the viral replication cycle. Localisation of NS1/2 to the ER might contribute to the formation of the membranous viral replication compartment, possibly by bridging the ER and replication membrane via its interaction with VAP and putative transmembrane domain (Baker et al., 2012).

### VAP-Exploiting Pathogens

#### Hepatitis C Virus

Hepatitis C virus (HCV) is an enveloped RNA virus that predominantly infects liver cells, and can cause liver cirrhosis and cancer. Upon HCV infection, a so-called membranous web, consisting primarily of double-membrane vesicles (DMVs), is formed, that is thought to be the site of viral RNA replication. Three HCV proteins have been reported to associate with VAP. While a direct interaction for the viral NS3/4A protease was not examined (Ramage et al., 2015), structural studies have looked into the binding domains of HCV proteins NS5A and NS5B. The viral RNA-dependent RNA polymerase NS5B interacts via its C-terminal auto-regulatory motif with the MSP domain of VAPA/B (Figure 2C) (Gupta and Song, 2016). This C-terminal motif seems to define whether NS5B is in a folded, auto-inhibitory state, or in a disordered, active state that binds to VAP and initiates RNA synthesis. Additionally, several studies report an interaction between HCV protein NS5A and VAPB, although they attribute the interaction to different domains. One study reveals that NS5A forms a dynamic complex with VAP-MSP by interacting via its disordered C-terminal D3 domain (Gupta et al., 2012). However, other studies report that the coiled-coil domain and transmembrane domain of VAPA/B and other residues of NS5A are essential for NS5A-VAP binding (Tu et al., 1999; Hamamoto et al., 2005; Goonawardane et al., 2017; Wang and Tai, 2019). Phosphorylation of NS5A has been reported to regulate the interaction with VAP (Evans et al., 2004; Goonawardane et al., 2017).

Overexpression and knockdown studies show that the VAP proteins play an important, but yet undefined role in the formation of the HCV replication complex and in RNA replication (Gao et al., 2004; Hamamoto et al., 2005). Although the function of NS5A/B-VAP binding in HCV infection is not fully understood, recent studies are starting to decipher how VAPA/B supports the viral replication. It has been suggested that VAP, NIR2 and OSBP operate in a phosphoinositide cycle between the ER and HCV DMV membrane (Figure 2C). Both VAP and the VAP-interactor NIR2 are required to upregulate phosphatidylinositol-4-phosphate (PI4P) levels during HCV infection (Wang and Tai, 2019), indicating that the phosphatidylinositol (PI) transfer protein NIR2 transfers PI from the ER membrane to the DMV membrane, which is then used to generate PI4P by phosphatidylinositol 4-kinase III α (PI4KA) (Berger et al., 2011). Interestingly, NS5A was shown to associate with and stimulate PI4KA activity. The PI4P is then exchanged for cholesterol from the ER by the VAP-interactor OSBP (Wang et al., 2014). The PI4P enrichment of the DMV membrane can also recruit other PI4P-interacting proteins to the DMV-ER MCS such as the VAP-interactor CERT, which transports ceramide from the ER to the DMV, where it can be converted to sphingomyelin, important for the biosynthesis of DMVs (Gewaid et al., 2020). NIR2, OSBP and CERT normally function at the Golgi-ER MCS (Table 1).

To inhibit the replication of HCV, the cell has mechanisms to disrupt the NS5A/B-VAPA/B binding. The ER-associated virus inhibitory protein Viperin, which binds to both NS5A and the C-terminal region of VAPA (Table 1), promotes the degradation of NS5A, an effect that is enhanced by VAPA (Wang et al., 2012; Ghosh et al., 2020). VAPC, an unstructured VAPB splice variant, acts as an endogenous inhibitor by binding to NS5B, interrupting the interaction of NS5B with VAPA/B (Kukihara et al., 2009; Goyal et al., 2012). The ability of VAPC to negatively regulate HCV replication has been of interest in anti-HCV drug development (Wen et al., 2011). Another potential anti-HCV drug also acts via disrupting the viral-host protein interaction; bicyclol restricts HCV replication by upregulating FFAT-motif containing protein GLTP (Table 1), which interrupted the interaction between VAPA and NS5A (Huang et al., 2019).

Overall, it seems that the VAP proteins anchor the viral RNA replication machinery to the ER membrane via viral NS5A/B interaction, and recruit host VAP interactors (e.g. NIR2, OSBP, CERT) for the synthesis of cholesterol and sphingomyelin, important for HCV replication. Targeting VAP in this way allows pathogens to use a single degenerate and potentially regulatable FFAT motif to interact with a range of useful host proteins.

#### Other Pathogens and Strategies for Utilisation of VAP

In addition to HCV, several other viruses hijack cholesterol trafficking within the cell. The Aichi virus (AiV) proteins 2B, 2BC, 2C, 3A, and 3AB are found in a complex with VAPA/B, OSBP and other components of the cholesterol transport machinery at Golgi-ER MCS such as the PI4P-phosphatase SAC1 and ACBD3 (which recruits PI4KB) (Sasaki et al., 2012; Ishikawa-Sasaki et al., 2018). The proteins are recruited to AiV genome replication sites at the replication organelle (RO)-ER MCS, where cholesterol accumulates in the RO membrane. Knockdown of each component resulted in inhibition of AiV RNA replication. Other viruses that utilise the OSBP-cholesterol transport to facilitate RNA synthesis at RO membranes include poliovirus (Arita, 2014), rhinovirus (Roulin et al., 2014) and encephalomyocarditis virus (EMCV) (Dorobantu et al., 2015). Although no virus-VAP (interactor) complexes have been reported for these viruses, poliovirus and EMCV proteins bind to PI4KB and PI4KA respectively, which stimulates PI4P production and leads to recruitment of OSBP.

The Adenovirus-2 (Ad2) adopts a different mechanism to employ the host cholesterol transport pathway. The Ad2 membrane protein RIDα directly interacts and recruits sterol-binding protein ORP1L to maturing early endosomes to form endosome-ER MCS via ORP1L-VAP binding (Cianciola et al., 2017) (Figure 2D). RIDα stabilizes the interaction between ORP1L and VAP, which supports the transport of cholesterol from maturing endosomes to the ER under high cholesterol conditions. The RIDα-ORPL1-VAP interaction induces the conversion of cholesterol into cholesteryl esters, which are stored in lipid droplets. This change in cholesterol trafficking attenuates proinflammatory TLR4 signalling involved in the innate immune response. ORP1L is also hijacked by the intracellular bacterium *Coxiella burnetii*, which forms a lysosome-like parasitophorous vacuole (PV) in the host cell for its replication (Justis et al., 2017). ORP1L is recruited to the PV by an unknown PV membrane protein, while also associating with ER-VAP. Although the function of ORP1L at PV-ER MCS in *C. burnetii* pathogenicity is unclear, ORP1L is important for PV expansion.

Herpes simplex virus type-1 (HSV-1) replicates its DNA and assembles its capsids in the host cell nucleus. The virion then crosses the nuclear envelope for further maturation in the cytoplasm. VAPB contributes to this nuclear egress as knockdown led to nuclear virion accumulation, however its exact role in this is still unclear (Saiz-Ros et al., 2019). VAP also plays a role in the replication of another DNA virus, the human papillomavirus 16 (HPV-16). However, instead of a role in nuclear egress, VAP is important in the nuclear entry pathway of HPV-16 (Siddiqa et al., 2018). Virus particles enter the cell via endocytic uptake, disassemble into protein complexes that traffic to the *trans*-Golgi-network (TGN) and then access the nucleus during mitosis when the nuclear envelope breaks down. VAP is required for the endosome-to-Golgi viral protein delivery, as it is essential for the formation of endosomal tubules induced upon HPV-16 infection. Whether these viruses exploit VAP directly via viral protein interactions or via other mechanism needs to be further elucidated.

The genetic disease cystic fibrosis (CF) is caused by mutations in the CF transmembrane conductance regulator (CFTR) protein. Patients have an increased susceptibility to bacterial infections such as *Pseudomonas aeruginosa* infection, which aggravates CF. *P. aeruginosa* exploits VAPB’s mitochondrial tethering function for infection (Rimessi et al., 2020). The bacteria induced increased VAPB and PTPIP51 expression in CF bronchial cells, but not in non-CF cells. The consequent increase in mitochondria-ER contacts caused impairment of autophagy, inducing inflammation and disease progression.

Overall, a variety of different pathogens utilise VAP interaction and modulation to allow them increased access to host resources. This likely reflects the multifunctionality of VAP as a versatile access point (Murphy and Levine, 2016) to the ER membrane and also the diversity of its interaction partners, which have roles in many different cellular functions. Therapeutic strategies which attempt to prevent pathogen access to VAP could perhaps be feasible but would need to be carefully targeted as inhibition of VAP function itself has a dramatic effect on cellular function and is linked to numerous neuronal disorders, as addressed in the following section.

## The Role of VAPB in Neuronal Disorders

VAPB has been linked to several neurological disorders, including amyotrophic lateral sclerosis (ALS), Alzheimer’s disease (AD) and the α-synucleinopathies, Parkinson’s disease (PD) and multiple system atrophy (MSA). This is via mutations in VAPB (ALS, PD) (Nishimura et al., 2004a; Kun-Rodrigues et al., 2015), disruption of VAPB’s interaction with PTPIP51 and hence ER-mitochondria contacts (ALS, AD, and PD via α-Synuclein binding, *see* Table 1) (Paillusson et al., 2017; Lau et al., 2020; Gómez-Suaga et al., 2022) or reduced VAPB levels (ALS, AD, MSA) (Anagnostou et al., 2010; Lau et al., 2020; Mori et al., 2021). Recent findings on the role of mutated VAPB in the pathogenesis of ALS are discussed in more detail below.

### Clinical Features of Amyotrophic Lateral Sclerosis Type 8 (ALS8)

An autosomal dominant missense mutation in VAPB, resulting in a substitution of proline to serine at codon 56 (P56S), was initially found in several Brazilian families (Nishimura et al., 2004a, 2004b; Marques et al., 2006). The patients presented with a heterogeneous phenotype of typical ALS, atypical ALS and late onset spinal muscular atrophy (SMA), and was termed ALS8 (OMIM 608627) (Nishimura et al., 2004a). Patients with ALS8 have predominant lower motor neuron involvement, with symptoms including progressive muscle weakness (mainly in the lower limbs), muscle atrophy, cramp, tremor, fasciculations, pain, abdominal protrusion, autonomic dysfunction (e.g. choking, constipation), and subtle cognitive and behavioural impairments (Nishimura et al., 2004a, 2004b; Marques et al., 2006; Funke et al., 2010; Kosac et al., 2013; Di et al., 2016; Chadi et al., 2017; Sun et al., 2017; Guber et al., 2018; de Alcântara et al., 2019; Trilico et al., 2020; Nunes Gonçalves et al., 2021; Temp et al., 2021; Leoni et al., 2022). ALS8’s clinical heterogeneity manifests not only in the symptoms but also in the age of onset (reported at 20–57 years) and the disease progression (rapid [<5 years] to slow [30+ years]). To understand the mechanisms behind this phenotypic variability, researchers compared gene expression profiles of iPSC (induced pluripotent stem cells)-derived motor neurons from mild and severe ALS8 patients (Oliveira et al., 2020). VAPB mRNA and protein levels were equally downregulated in mild and severe patients. The differentially expressed genes found in the study were associated with pathways involved in protein translation and protein targeting to the ER; pathways that may mitigate neurodegeneration in the mild ALS8 patients by maintaining proteostasis. Interestingly, a reduction in VAPB mRNA and protein levels was also observed in the spinal cord of sporadic and familiar (superoxide dismutase 1 (SOD1)-linked) ALS patients and mice, suggesting a role of VAPB in the pathogenesis of non-VAPB linked ALS as well (Teuling et al., 2007; Anagnostou et al., 2010). These reduced levels might be associated with SNPs (single-nucleotide polymorphisms) within the VAPB gene (Chen et al., 2010). It has even been suggested that VAPB aggregates can be used as a pathologic marker in the screening of sporadic non-VAPB linked ALS, as VAPB clusters were detected in peripheral blood mononuclear cells (PBMCs) and fibroblasts isolated from these patients (Cadoni et al., 2020).

Haplotype analysis showed a common Portuguese ancestor of the Brazilian families, with a founding event 23 generations ago, resulting in about 200 affected family members (Nishimura et al., 2005). Mutations in VAPB have not been associated with sporadic ALS (Conforti et al., 2006; Kirby et al., 2007) and the frequency of VAPB mutations is low in other populations (Tsai et al., 2011; Ingre et al., 2013; Kenna et al., 2013). However, the P56S mutation has also been identified in German, Japanese, Chinese and North American families displaying ALS8 symptoms, and have arisen independently from the Brazilian patients (Funke et al., 2010; Millecamps et al., 2010; Di et al., 2016; Guber et al., 2018). Another mutation in codon 56 of VAPB, in which proline is substituted for histidine (P56H), has also been found, in a Chinese family with similar clinical features as patients with P56S (Sun et al., 2017). Other mutations located in VAPB and associated with ALS are T46I, A145V and V234I (see *Other Mutations in VAPB*) (Chen et al., 2010; van Blitterswijk et al., 2012; Kabashi et al., 2013).

Although VAPB is ubiquitously expressed in the body and fulfils functions important for basal cell performance, it is mainly motor neuron dysfunction that is reported in the VAPB P56S/H patients. Electromyography and muscle/nerve biopsies revealed neurogenic damage with chronic denervation of muscles and reduced numbers of myelinated axons (Nishimura et al., 2004a; Marques et al., 2006; Kosac et al., 2013; Di et al., 2016; Sun et al., 2017; Guo et al., 2020). Additionally, neuroanatomical abnormalities were observed in ALS8 patients, including atrophy in the brainstem, globi pallida and upper cervical spinal cord (Leoni et al., 2022). The reason why VAPB mutations lead specifically to neurodegeneration is not well understood, although VAPB has been found to be highly abundant in motor neurons and different regions of the brain (Teuling et al., 2007; Larroquette et al., 2015; Leoni et al., 2022).

### VAPB Aggregates

#### VAPB Aggregate Features and Formation

Several studies have reported that overexpression of VAPB P56S induces the formation of insoluble cytosolic aggregates in neuronal and non-neuronal cells (Nishimura et al., 2004b; Kanekura et al., 2006; Teuling et al., 2007), in culture as well as in transgenic mice and *Drosophila* ALS models (Chai et al., 2008; Ratnaparkhi et al., 2008; Qiu et al., 2013). The aggregation-prone VAPB P56S recruits wild-type VAPB and, to a lesser extent, VAPA to the aggregates, having a dominant-negative effect on normal VAP function (Kanekura et al., 2006; Teuling et al., 2007; Chai et al., 2008; Ratnaparkhi et al., 2008; Suzuki et al., 2009). The VAPB mutant has also been shown to sequesters ER-Golgi recycling protein YIF1A (via its TMD) to the aggregates, depleting the protein from these organelles (Kuijpers et al., 2013b). Nevertheless, VAPB P56S does not seem to induce “classical protein aggregates,” formed of insoluble fibrils, a hallmark of other neurodegenerative disorders like Huntington’s disease (huntingtin), PD (α-synuclein) and ALS (SOD1). For example, VAPB P56S forms aggregates rapidly after expression (<2 h), while the formation of SOD1 aggregates takes hours to days (Matsumoto et al., 2005; Fasana et al., 2010). Additionally, ultrastructural studies showed that overexpression of the mutant VAPB protein caused accumulation of large membranous aggregates, consisting of ribbons of stacked ER cisternae (Teuling et al., 2007; Fasana et al., 2010; Papiani et al., 2012). Live cell photobleaching experiments, using ER membrane-targeted GFP, revealed that VAPB P56S-ER subdomain inclusions are continuous with the rest of the ER (Fasana et al., 2010). However, there is some discrepancy between overexpression studies about the presence of proteins from the secretory pathway in the VAPB aggregates; for instance, ER luminal proteins calreticulin and PDI, and ER membrane protein calnexin associate with mutant VAPB aggregates is some studies, whilst others observed exclusion of these proteins (Kanekura et al., 2006; Teuling et al., 2007; Prosser et al., 2008; Fasana et al., 2010; Kuijpers et al., 2013a). This may be attributed to differences in cell lines, VAPB expression levels, and the exclusion of some (rough) ER membrane proteins from the aggregates (Fasana et al., 2010).

To understand how VAPB P56S induces aggregate formation, we will first discuss how the mutation affects the protein structure. VAPB proline 56 is conserved in VAPA, but mutating this residue does not seem to have such a significant effect, with some studies suggesting that no aggregation was observed whilst others observe minor levels of aberrant aggregation for VAPA P56S, notably in HeLa cells (Teuling et al., 2007; Prosser et al., 2008; Suzuki et al., 2009). VAPA P56S’s resistance to aggregation seems to rely on two other proline residues present in this region, whereas VAPB P56S has only one remaining proline residue (Nakamichi et al., 2011). Substituting one of the prolines in VAPA P56S to the equivalent in VAPB P56S (VAPA P56S/P63A), resulted in the formation of membranous aggregates indistinguishable from those observed with VAPB P56S. The three proline residues of VAPA are conserved in the yeast VAP protein Scs2p, which is also resistant to the ALS8-causing mutation, showing that the proline distribution is an important feature in the pathophysiology of ALS8 (Nakamichi et al., 2011).

**FIGURE 3:**
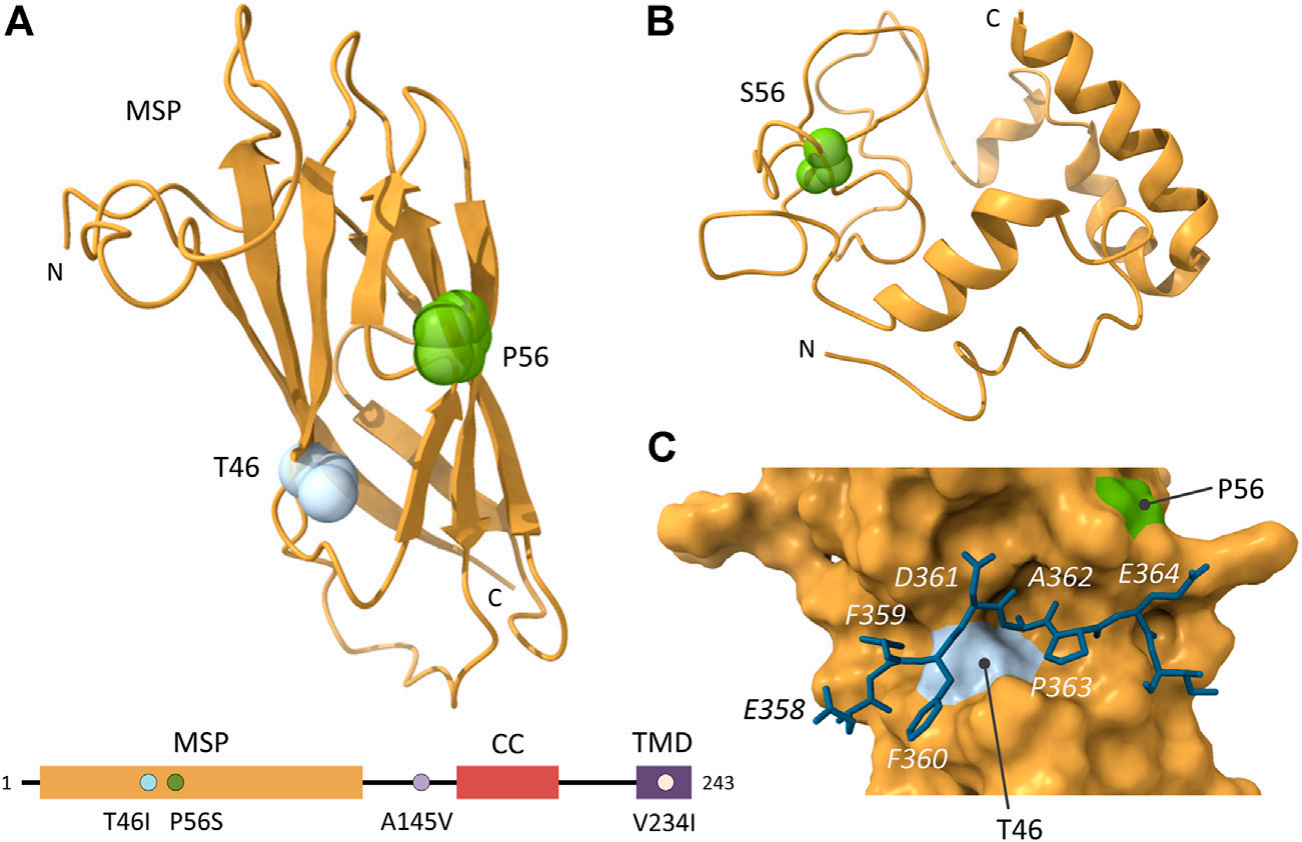
Structure of the VAP MSP domain. **(A)** Structure of the MSP domain of VAPB (PDB ID: 3IKK) and schematic representation of the domain architecture of VAPB, with the ALS-related mutations indicated. The two ALS-related residues that are located in the MSP domain (T46 and P56) are mapped onto the structure. **(B)** Structure of the MSP domain of VAPB P56S (PDB ID: 2MDK). The ALS-related mutation S56 is mapped onto the structure. **(C)** Structure of the MSP domain of VAPA in complex with the OSBP FFAT motif (_358_EFFDAPE I) (PDB ID: 2RR3). MSP residues T46 and P56 are indicated. The FFAT core residues of OSBP are written in *italic*. Images created with UCSF ChimeraX (Pettersen et al., 2021).

P56 is located in the MSP domain of VAPB and is critical for the correct folding of the seven β-strands of the MSP domain (Figure 3A) (Shi et al., 2010). P56 stabilizes the *cis*-peptide bond within the S-shaped loop that connects strands D1 and D2 (Kaiser et al., 2005; Teuling et al., 2007; Shi et al., 2010). The P56S mutation induces a conformational change within the recombinant MSP domain, resulting in the exposure of hydrophobic patches, which may enhance oligomerization of the mutant VAPB protein under physiological conditions (Figure 3B) (Kim et al., 2010). However, studies with recombinant MSP P56S domains show differences in structural stability and solubility (Kim et al., 2010; Shi et al., 2010). P56S eliminates the native β-sheet structure in water, and the exposed hydrophobic patches seem to drive aggregation of recombinant MSP P56S, making the structure highly insoluble in various buffers (Shi et al., 2010; Qin et al., 2013a). This makes it difficult to understand exactly how the VAPB P56S structure behaves under physiological conditions. Nevertheless, it has been shown that MSP P56S retains its ability to bind to FFAT motif-containing proteins in HeLa cells, but the FFAT-binding of full-length VAPB P56S is perturbed (Kim et al., 2010). The aberrant oligomerization of full-length VAPB P56S may interfere with the binding of FFAT motifs to the MSP domain. In line with this, no FFAT-motif containing proteins were observed in pull-down assays using biotinylation-tagged VAPB P56S (Teuling et al., 2007). However, overexpression of a FFAT motif peptide rescued the aggregation phenotype of the mutant, suggesting protein stabilisation via FFAT motif-binding (Prosser et al., 2008). Additionally, VAPB P56S induces clustering of mitochondria and peroxisomes that colocalise with the VAPB aggregates (De Vos et al., 2012; Hua et al., 2017). The clustering of peroxisomes was dependent on the presence of ACBD5, suggesting that the mutant VAPB can sequester FFAT-motif containing proteins such as peroxisomal ACBD5 and possibly mitochondrial PTPIP51 (De Vos et al., 2012; Hua et al., 2017). However, FFAT-proteins ORP9 and NIR2 were not detectable in the VAPB aggregates (Kuijpers et al., 2013a). In summary, the P56S mutation causes conformational changes in the MSP domain and although this does not affect FFAT-binding to the domain on its own, in the presence of full-length VAPB, exposed hydrophobic patches cause enhanced oligomerization of the protein, which seems to reduce accessibility to the FFAT-binding site.

The disordered MSP P56S domain, but not the wild-type MSP domain, is able to interact with dodecylphosphocholine, a lipid commonly used to resemble membrane lipids, transforming the domain into a highly helical conformation (Qin et al., 2013b). This allows MSP P56S to be inserted into membrane environments (Qin et al., 2013a). Therefore, the interaction of VAPB P56S with lipids from the ER membranes could provide a mechanism for the formation of the membranous aggregates. The presence of membrane structures within the aggregates could also be attributed to VAPB being a tail-anchored protein. VAPB P56S has been shown to be efficiently post-translationally inserted into the ER membrane, after which it rapidly clusters (Fasana et al., 2010). This is further confirmed by a study showing that co-expression of a FFAT-containing peptide with VAPB P56S, partially restored the characteristic reticular ER pattern of VAPB (Prosser et al., 2008), suggesting that FFAT binding can maybe stabilise the mutant MSP structure and that the MSP domain/FFAT-interaction plays an important role in the formation of (membranous) aggregates.

Overall, VAPB P56S seems prone to aggregation due to instability of its MSP structure caused by the mutation. Because of its unaffected TMD, the mutant protein is still targeted to the ER membrane (Fasana et al., 2010), where it forms clusters, recruits wild-type VAPA/B and VAP interactors, and induces the formation of membranous clusters (Teuling et al., 2007; Fasana et al., 2010). Conceivably, newly synthesized mutant VAPB could also aggregate in the cytosol before its insertion into the ER membrane. This may depend on the rate of protein synthesis and levels/capacity of the chaperone machinery, which may differ between different cell types, but could then give rise to two different types of aggregates, cytosolic and membranous, which may explain some discrepancy between studies.

#### VAPB Aggregate Clearance

While overexpression studies show that VAPB P56S is aggregation prone, aggregate formation was also induced at physiological conditions at low levels of mutant VAPB, comparable to endogenous wild-type protein, in HeLa cells (Fasana et al., 2010; Papiani et al., 2012). However, more research is required to clarify in what extend these aggregates form in patients; it has been shown that iPSC-derived motor neurons from ALS8 patients have reduced levels of VAPB and no signs of aggregate accumulation (Mitne-Neto et al., 2011; Oliveira et al., 2020), while ALS8 patient-derived muscle biopsy and fibroblasts revealed VAPB aggregates (Tripathi et al., 2021). As HeLa cells also displayed aggregated VAPA P56S (Teuling et al., 2007), which was not observed in other cell types, it seems likely that different cell types show altered VAP aggregate accumulation.

Discrepancy in detection of aggregates in patients might be due to differences in clearance of mutant VAPB. VAPB P56S has been reported to be less stable than the wild-type protein in both cultured cells and transgenic mice (Papiani et al., 2012; Aliaga et al., 2013; Genevini et al., 2014). VAPB P56S was polyubiquitinated shortly after synthesis and degraded by the proteasome in inducibly-expressing HeLa and NSC34 (motoneuronal) cells, with no evident involvement of basal autophagy (although it can be targeted by stimulated autophagy) (Kanekura et al., 2006; Papiani et al., 2012; Genevini et al., 2014). Ubiquitination of VAPB P56S has also been observed in motor neurons and muscle of transgenic mice and flies (Ratnaparkhi et al., 2008; Tsuda et al., 2008; Tudor et al., 2010). The data further indicates that in the HeLa cells and transgenic mice, the mutant protein initially avoids degradation, clusters and is then cleared by the proteasome (Papiani et al., 2012; Kuijpers et al., 2013a). This comprises the involvement of ER membrane chaperone BAP31 and the ATPase chaperone p97/VCP, proteins involved in ER-associated protein degradation (ERAD), likely by extracting mutant VAPB from the ER membrane. However, a study reported that overexpression of both wild-type and mutant VAPB impaired proteasome activity, possibly by inducing ER stress (see below) (Moumen et al., 2011), although this might be attributed to the high levels of expressed VAPB in comparison to the inducible system. Interestingly, it has also been reported that VAPB P56S is resistant to proteolysis by an unidentified protease that releases the MSP domain from wild-type VAPB (Gkogkas et al., 2011).

### Disruption of Cellular Homeostasis

Below we highlight some of the functions of VAPB and the effects that VAPB P56S has on ER stress responses and autophagy. But since VAPB has many functions and binding partners (*see* Table 1), and the P56S mutation impacts the protein properties (*see* above), it is plausible that most processes involving VAP are in some extent impacted by mutant VAPB, including organelle tethering (Yamanaka et al., 2020) and regulation of PI4P levels (Wilson et al., 2021). We focus on how VAPB P56S affects motor neurons specifically. However, in addition to neurological problems, ALS8 patients also exhibit altered metabolic functions, such as dyslipidemia with increased cholesterol and triglyceride levels (Marques et al., 2006). VAPB P56S was found to suppresses adipocyte differentiation (Tokutake et al., 2015a) and VAPB is involved in different cholesterol and triglyceride pathways via its binding partners, as shown in Table 1.

#### ER Stress

The P56S mutant VAPB causes ER stress (Aliaga et al., 2013; Larroquette et al., 2015), altered ER domain properties (Fasana et al., 2010; Papiani et al., 2012; Yamanaka et al., 2020) and malfunction of the unfolded protein response (UPR), a physiological reaction to suppress accumulation of misfolded proteins in the ER (Kanekura et al., 2006; Suzuki et al., 2009). In mammalian cells, the three main signalling pathways of UPR are IRE1, ATF6, and PERK–with all three shown to be affected by mutant VAPB. VAPB P56S suppress the IRE1-XBP1 pathway that activates expression of UPR target genes, such as chaperones and ERAD components (Kanekura et al., 2006; Suzuki et al., 2009; Tokutake et al., 2015b). VAPB directly interacts with the ER-localized transcription factor ATF6 which, by acting as an ER stress sensor, regulates the transcription of genes encoding chaperones and other UPR transcription factors (Gkogkas et al., 2008). VAPB P56S was shown to attenuate the ATF6-mediated UPR transcription. On the other hand, VAPB P56S activates UPR via PERK-ATF4 which, by promoting the expression of the pro-apoptotic gene CHOP, initiates the cell apoptotic pathway under prolonged ER stress (Aliaga et al., 2013; Tokutake et al., 2015a). Increased basal ER stress and UPR activation has also been reported in the ALS8 patient-derived fibroblasts (Guber et al., 2018). Overall, if the UPR impacted by VAPB P56S cannot restore proteostasis, it might lead to apoptosis.

#### Autophagy

Mutant VAPB has been linked with dysfunctional autophagy (Zhao et al., 2018; Tripathi et al., 2021). The P56S mutation reduced VAPB’s interaction with early autophagy proteins ULK1 and FIP200, impairing autophagosome biogenesis (*see* Table 1) (Zhao et al., 2018). Additionally, VAPB P56S accumulates in autophagosomes and impairs their clearance, showing that VAPB acts at different stages of autophagy (Larroquette et al., 2015; Tripathi et al., 2021). An accumulation and sequestering of autophagic markers p62 and LC3 at VAPB P56S aggregates was also observed in ALS8 patient fibroblasts and muscle biopsies (Tripathi et al., 2021). Impairment of autophagy by mutant VAPB can result in the aggregation of FUS, TDP-43 and Matrin 3 – mutations in which are associated with familial ALS–leading to the formation of stress granules (Tudor et al., 2010; Tripathi et al., 2021). Overexpression of FUS and TDP-43 have both been linked with disruption of the PTPIP51-VAPB association and hence, mitochondria-ER contacts (Stoica et al., 2014, 2016) (Figure 1). Loosening mitochondria-ER contacts via PTPIP51 or VAPB knockdown has been shown to stimulate autophagosome formation by disrupting the Ca^2+^ delivery to mitochondria from ER stores (Gómez-Suaga et al., 2017). VAPB P56S and TDP-43 may also co-operate in the pathogenesis of ALS by activating the mitochondrial apoptotic pathway (Suzuki and Matsuoka, 2011). VAPB is also involved in ER-phagy, a selective form of autophagy for degradation of the ER, via interaction with the soluble ER-phagy receptor CALCOCO1 which, via ATG8 binding, connects the ER and autophagosome membranes (*see* Table 1) (Nthiga et al., 2020).

#### VAP in Neurones

Although VAPB is ubiquitously expressed and hence disruption caused by the P56S mutation would affect all cells in the body, ALS8 patients mainly present with (lower) motor neuron dysfunction and neurodegeneration. The large size and complex morphology of motor neurons make the maintenance of protein homeostasis and the distribution of organelles a greater challenge. Hence, motor neurons may be more vulnerable to the overall homeostatic disruption caused by aberrant VAPB. Several studies illustrate how VAPB P56S can affect neuron-specific processes and morphology. For instance, the mutant VAPB disrupts anterograde mitochondrial axonal transport by disrupting Ca^2+^ homeostasis in neurons (Mórotz et al., 2012). Peroxisomal movement in hippocampal neurones has also been shown to resemble that of mitochondria and be altered by levels of the peroxisome-ER tethering protein ACBD5 (Wang et al., 2018). However, unlike for mitochondria, this did not appear to be dependent upon VAPB interaction. A loss of the VAPB orthologue in *Drosophila* also resulted in abnormal organelle distribution in neuronal axons and dendrites, including mitochondria and the Golgi apparatus, which may have contributed to the altered dendrite morphology (Kamemura et al., 2021), indicating the importance of ER-tethering in organelle distribution. Furthermore, mitochondria-ER contacts, mediated by the PTPIP51-VAPB interaction, are present at synapses and regulate synaptic function (Gómez-Suaga et al., 2019). Loss of PTPIP51 or VAPB reduced synaptic function and altered dendritic morphology. VAPB P56S also sequesters VAP-interactor YIF1A (Table 1), which regulates membrane trafficking into dendrites and dendritic morphology (Kuijpers et al., 2013b). VAPB is also important for neurite extension of motor neurons (Genevini et al., 2014), possibly via its interaction with protrudin (Table 1) (Saita et al., 2009). Additionally, VAPB P56S led to a loss of HCN channel activity, important for neuronal and cardiac pacemaker currents (Silbernagel et al., 2018). These alterations in motor neurons may partly explain the neurodegeneration and muscle-related symptoms observed in ALS8 patients. VAPB P56S may also affect muscle cells more directly; the VAPB mutation disrupted the formation of multinuclear myotubes (muscle fibres) by mouse skeletal muscle cells (Tokutake et al., 2015b) and caused accumulation of ER Ca^2+^ sensor STIM1 at neuromuscular junctions (NMJ) in muscle fibres of ALS8 patients, suggesting altered intracellular Ca^2+^ homeostasis (Goswami et al., 2015). Interestingly, in *Drosophila*, VAPB regulates the number and size of synaptic boutons at NMJ (Pennetta et al., 2002; Chai et al., 2008). Additionally, VAPB deficient mice showed abnormal skeletal muscle energy metabolism upon fasting (Han et al., 2013). Impaired degradation pathways, accumulation/aggregation of misfolded proteins and disrupted Ca^2+^ homeostasis in motor neurons and muscle fibres may all contribute to ALS8 pathogenesis.

Acknowledging the various roles VAPB plays in many important physiological pathways, it is not surprising that disruption of the protein has a major effect on cellular homeostasis. Nevertheless, it is still under debate whether the P56S mutation in VAPB induces the symptoms of ALS8 patients by a loss of function (lost/reduced protein interactions), a toxic gain of function (aggregate formation, protein sequestering), or a dominant negative effect (wild-type VAP recruitment). VAPB P56S aggregates in the nervous system of transgenic mice did not cause motor neuron dysfunction, suggesting that aggregates are not sufficient to initiate pathogenesis (Tudor et al., 2010; Qiu et al., 2013), although, with a higher fold increase of VAPB P56S protein expression, mice developed abnormal motor behaviour and progressive degeneration of corticospinal motor neurons (Aliaga et al., 2013). A study using both homozygous and heterozygous VAPB P56S knock-in mice showed defects in motor behaviours, with accumulation of cytoplasmic inclusions selectively in motor neurons before onset of the defects, though the homozygous knock-in mice presented with a more severe phenotype, reflecting a dose-dependent effect of the mutant protein (Larroquette et al., 2015). On the other hand, VAPB knockdown was sufficient to lead to motor deficits in zebrafish and mild, late-onset motor deficits were observed in VAPB knockout mice, however, VAPB depletion was unable to induce a complete ALS phenotype (Kabashi et al., 2013). Thus, VAPB P56S abnormalities might be a combination of gained and lost functions, in a dominant and dose-dependent manner.

### Other Mutations in VAPB

A second mutation located in the MSP domain of VAPB has also been associated with familial ALS. An amino acid change from threonine to isoleucine at codon 46 (T46I) was identified in a patient from the United Kingdom, with non-Brazilian kindred–affected family members were not available to screen (Chen et al., 2010). The patient presented with typical ALS, with onset of symptoms at the age of 73 years. Unlike the P65S mutation that completely eliminates the native MSP structure in various buffers (Shi et al., 2010), MSP T46I retains a structure highly similar to the native MSP domain, although with reduced stability (Lua et al., 2011). This makes the MSP domain more easily accessible to unfolded intermediates that are prone to aggregation as shown *in vitro*, in cultured cells as well as *in vivo* (Chen et al., 2010; Lua et al., 2011). T46 is part of the hydrophobic pocket that binds the side chain of FFAT motif residue 5 (A) and forms hydrogen bonds with the side chains of FFAT motif residues 2 (F) and 3 (F) (Figure 3C) (Kaiser et al., 2005; Furuita et al., 2010). The threonine to isoleucine substitution induced some dynamic changes of local regions within the MSP domain (Lua et al., 2011). These alterations seem to affect the ability of VAPB to bind FFAT-motif containing proteins, as illustrated with the NIR2 FFAT motif that showed a 3-fold decrease in binding affinity (Chen et al., 2010). Analysis of VAPB T46I in neuronal cells and *D. melanogaster* indicates similar cellular abnormalities as with the P56S mutation, such as wild-type VAPB sequestering, ER fragmentation and neurodegeneration.

Two VAPB mutations outside of the MSP domain have also been identified in ALS patients. An alanine to valine substitution at codon 145 (A145V) was identified (Kabashi et al., 2013), which is located in the region between the MSP and TMD of VAPB but little else is known about the pathogenicity of A145V. Furthermore, V234I was identified in a patient of Dutch origin, who also harboured a repeat expansion in C9orf72, an ALS causative gene (van Blitterswijk et al., 2012). Transgenic expression of the VAPB V234I orthologue in *D. melanogaster* was able to induce ALS hallmarks (Sanhueza et al., 2014). The valine to isoleucine substitution is located in the transmembrane domain of VAPB, and although it is close to the dimerization motif, it did not affect VAPB dimerization (Chattopadhyay and Sengupta, 2014). However, the V234I mutation seems to affect the ER-targeting of VAPB as it did not localize with ER-marker PDI. The V234I mutated VAPB did not form typical P56S aggregates, but formed small aggregates/granules in HeLa cells, which may lead to cell death (Chattopadhyay and Sengupta, 2014).

## Conclusion

Here, we provided a timely summary of the constantly growing number of VAP interacting proteins, their FFAT motifs (if present) and interaction domains, which will present a helpful overview for future studies on VAP binding partners. We discussed new findings on the regulation of VAP binding by phosphorylation of the FFAT motif core, and the role of GSK3β in the regulation of both mitochondria-ER and peroxisome-ER membrane contact sites. How the interaction of VAP with tether proteins and other interaction partners is regulated, is still not well explored. Future studies may shed light on the regulation of those interactions and their impact on the multiple cellular functions of VAP proteins. An intriguing aspect is also the hijacking of VAP by bacteria and viruses and its role in pathogen infection. It will be interesting to investigate if and how the organelle-specific binding partners are influenced, and if those proteins are suitable new therapeutic targets to combat pathogen infection. Furthermore, the impact of VAP mutations on neurological disorders deserves further investigation. Although our knowledge about VAP and its binding partners at membrane contacts has increased, we do not yet fully understand the (patho)physiological consequences of altered ER-organelle contacts and how this would impact on neurological functions. Thus, VAP proteins and their interacting proteins will remain in the focus of fundamental, discovery-based research as well as biomedical studies.

## Data Availability

All datasets generated for this study are included in the article.

## Abbreviations

ALS, amyotrophic lateral sclerosis; FFAT, two phenylalanines (FF) in an acidic tract; MCS, membrane contact sites; MSP, major sperm protein; PI4P, phosphatidylinositol-4-phosphate; TMD, transmembrane domain; VAP, vesicle-associated membrane protein (VAMP)-associated protein.
